# Supplementary material for: Comparison of the Blood–Brain Barrier Transport and Vulnerability to P-Glycoprotein-Mediated Drug–Drug Interaction of Domperidone versus Metoclopramide Assessed Using In Vitro Assay and PET Imaging
Source: Pharmaceutics. 2022 Aug 9;14(8):1658. doi: 10.3390/pharmaceutics14081658 (PMC9412994; doi:10.3390/pharmaceutics14081658)
Supplement: Supplementary file 1 [file pharmaceutics-14-01658-s001.zip › pharmaceutics-1830752-supplementary.pdf]

Article

Comparison of the blood-brain barrier transport and vulnerability to P-glycoprotein-mediated drug-drug interaction of domperidone versus metoclopramide assessed using in vitro assay and PET imaging.

Louise Breuil, Sébastien Goutal, Solène Marie, Antonio Del Vecchio, Davide Audisio, Amélie Soyer, Maud Goislard, Wadad Saba, Nicolas Tournier, Fabien Caillé

Supplementary Material

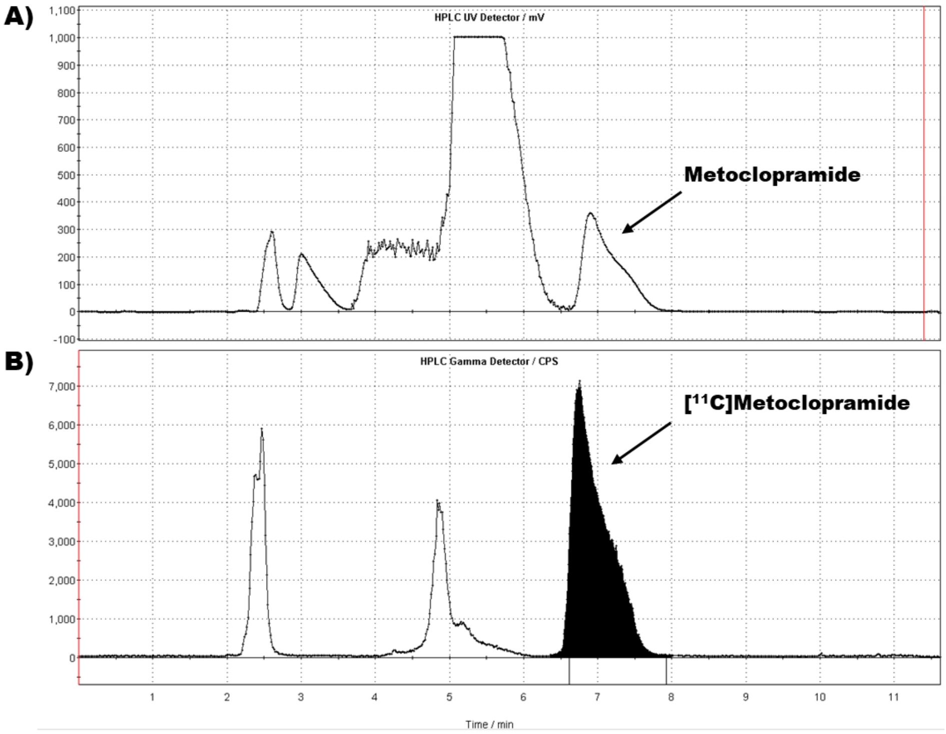

Figure S1. Reverse phase semi-preparative HPLC purification of [^{11}C]metoclopramide. A) UV chromatogram at λ = 220 nm; B) Gamma chromatogram.

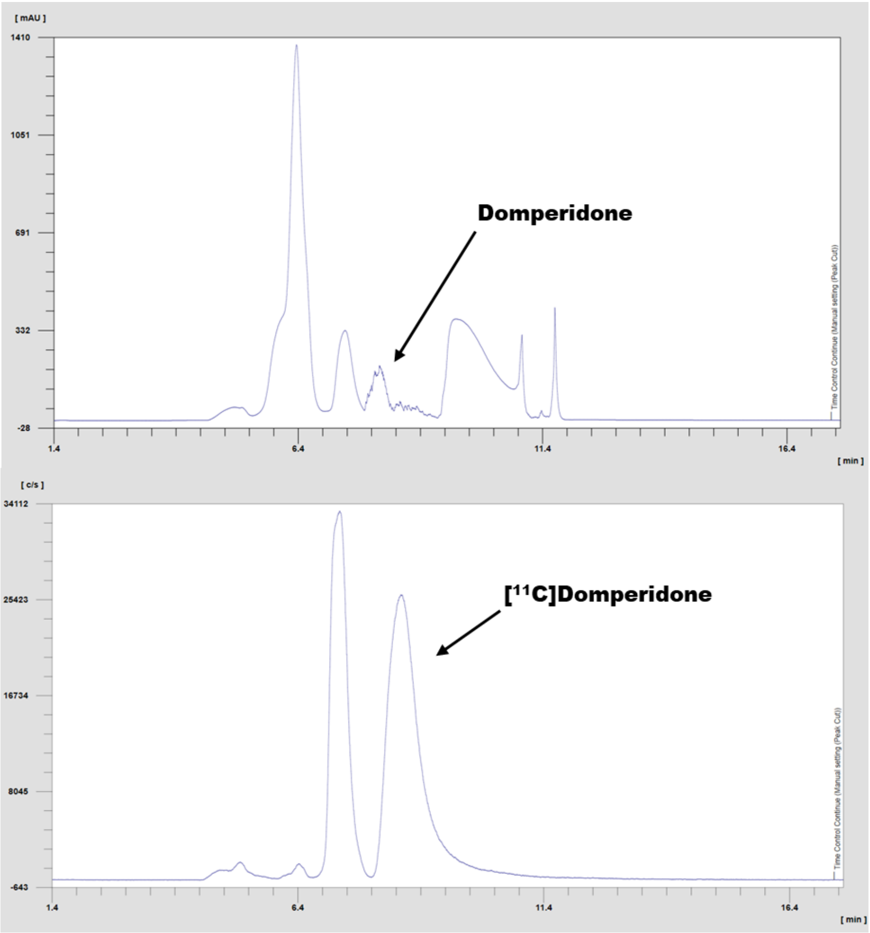

Figure S2. Reverse phase semi-preparative HPLC purification of [^{11}C]domperidone. A) UV chromatogram at λ = 280 nm; B) Gamma chromatogram.

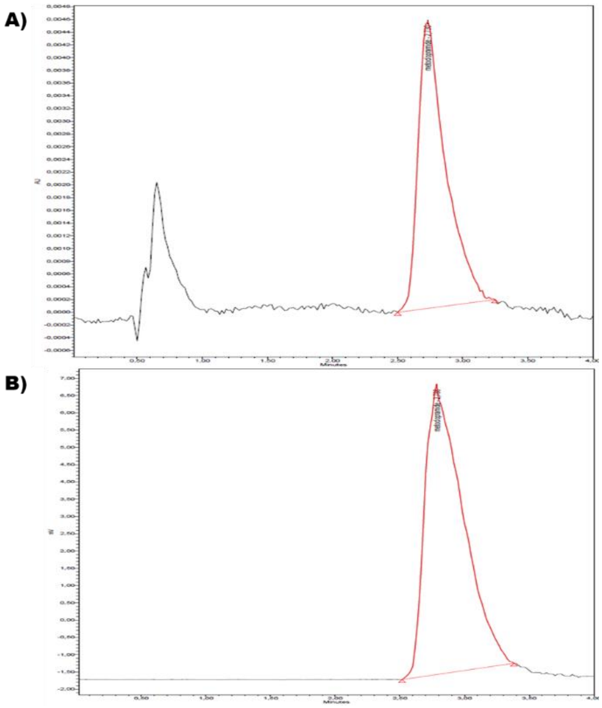

Figure S3. Quality control of [^{11}C]metoclopramide by reverse phase analytical HPLC. A) UV chromatogram at λ = 274 nm; B) Gamma chromatogram.

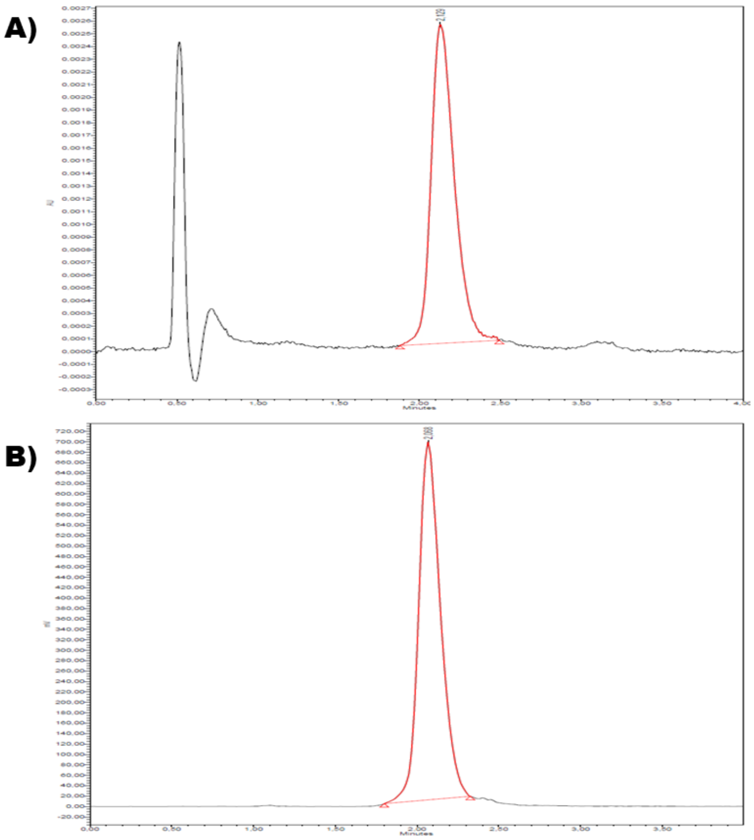

Figure S4. Quality control of [^{11}C]domperidone by reverse phase analytical HPLC. A) UV chromatogram at λ = 285 nm; B) Gamma chromatogram.
